# Feasibility and safety of surgical microwave ablation for hepatocellular carcinoma in elderly patients: a single center analysis in Japan

**DOI:** 10.1038/s41598-020-71095-7

**Published:** 2020-08-26

**Authors:** Hajime Imamura, Yuko Takami, Tomoki Ryu, Yoshiyuki Wada, Shin Sasaki, Hiroki Ureshino, Hideki Saitsu

**Affiliations:** Department of Hepato-Biliary-Pancreatic Surgery, Clinical Research Institute, National Hospital Organization Kyushu Medical Center, 1-8-1, Jigyohama, Chuo-ku, Fukuoka city, Fukuoka 810-8563 Japan

**Keywords:** Hepatocellular carcinoma, Surgical oncology

## Abstract

The feasibility and safety of microwave ablation in elderly hepatocellular carcinoma (HCC) patients remains unknown. The aim of this study was to evaluate the feasibility and safety of surgical microwave ablation for HCC in patients older than 80 years of age. This retrospective study enrolled consecutive 114 patients older than 80 years of age who underwent surgical microwave ablation for HCC between July 1994 and December 2017. We analyzed perioperative outcomes and long-term outcomes to clarify the prognostic factors. The 1-, 3-, 5-year overall survival and recurrence-free survival rates were 97.3%, 76.0%, 49.2% and 84.2%, 44.7%, and 32.5%, respectively. The overall major morbidity rates (Clavien–Dindo grade IIIA or above) were 2.6%. There were no cases of mortality. Multivariate analysis showed that hepatitis C virus antibody (HCV-Ab) positivity and the presence of multiple tumors were independent prognostic factors for long-term outcomes. The overall survival rate of patients with HCV-Ab negative and single tumor was better than that of other patients (*p* = 0.026). Surgical microwave ablation was feasible and safe for elderly patients with HCC. Elderly patients with HCV-Ab negative and single tumor would be expected to have better long-term outcomes after surgical microwave ablation.

## Introduction

With the population aging because of improved medication and healthcare, management of malignant diseases in elderly patients is becoming a major topic of interest worldwide. In Japan, an increased proportion of elderly patients with hepatocellular carcinoma (HCC) has been reported according to Japanese nationwide surveys of primary liver cancer^1^.

Hepatic resection is generally considered as the first-line treatment for HCC patients. Some studies have shown the feasibility and safety of hepatic resection for elderly patients with HCC^2–4^. However, a previous report has revealed that hepatic resection is performed for only 31% of elderly HCC patients aged > 75 years^2^. The indication of hepatic resection is limited because of comorbidity or a poor general status of elderly patients.

Radiofrequency ablation (RFA) and microwave ablation have received recognition as alternative treatment strategy as local ablation therapy for HCC treatment^5–8^. Among these local ablation therapies, the efficacy and safety of RFA have been reported in elderly patients with HCC^5,9^. Microwave ablation has been used as another thermal ablative technique for HCC treatment^6,10^. Several recent studies have reported that microwave ablation is an effective treatment for HCC^11–14^. We have performed surgical microwave ablation, termed microwave coagulo-necrotic therapy (MCN), not only for primary HCC but also for recurrent HCC since 1988, and reported its feasibility, safety and oncological long-term outcomes previously^14–17^. However, the feasibility and safety of microwave ablation in elderly HCC patients remains unknown.

The present study aimed to investigate the feasibility and safety of surgical microwave ablation for HCC in patients older than 80 years of age and clarify the prognostic factors.

## Patients and methods

### Patients and diagnosis

Between July 1994 and December 2017, 986 patients underwent MCN for primary HCC at our department. Among them, data from consecutive patients over 80 years of age were collected. We then investigated patient characteristics, perioperative outcomes, and long-term outcomes.

We preoperatively diagnosed HCC lesions in each patient by using imaging modalities (such as ultrasonography, dynamic computed tomography, and/or enhanced magnetic resonance imaging). In all patients, the final diagnosis of HCC was confirmed by pathological examination of a tumor biopsy.

This study was conducted in accordance with the Declaration of Helsinki and the ethical guidelines for clinical studies of the Ministry of Health, Labor, and Welfare of Japan. The study protocol was approved by the Ethics Committee on Clinical Investigations of Kyushu Medical Center (Approval No. 19C128).

### Preoperative clinical evaluation

The recorded data included age, sex, American Society of Anesthesiologists (ASA) physical status classification, concurrent disease (hypertension, diabetes mellitus, dementia, cardiovascular disease, cerebrovascular disease, renal disease, and respiratory disease), past history of malignant disease, hepatitis virus markers [hepatitis B virus surface antigen (HBs-Ag) and HCV-Ab], blood chemistry parameters, indocyanine green retention rate at 15 min (ICG-R15), Child–Pugh classification, tumor markers [alpha-fetoprotein (AFP), des-γ-carboxy prothrombin (DCP)], tumor size, and number of tumors. Serum concentrations of AFP and DCP were measured within 1 week before treatment. The upper limits of the normal ranges of AFP and DCP at our institution were 20 ng/mL and 40 mAU/mL, respectively.

### Treatment and patient follow-up

MCN procedures were performed according to our standardized methods as reported previously^14,18^. We performed MCN by using an open approach (laparotomy, thoracotomy, or laparoscopy) in all patients. MCN initially ablates around the tissue of tumor and then ablates center of the tumor to prevent spreading of cancer cells. During the operation, we perform routine intraoperative ultrasound to identify lesions and monitor the treatment effects. The surgical indication was decided at HCC team meetings comprehensively by evaluating each patient’s liver functions, tumor location, and extent of tumor spread. MCN was considered to treat patients with HCC ≤ 3 cm preferentially. In patients with impaired liver functions, older age, a poor performance status, or who declined hepatic resection of HCC > 3 cm, MCN was also selected as one of the treatment strategies. As to the number of nodules, we usually attempted microwave ablation for five or fewer tumors. In case of more than five nodules, MCN also attempted if we decided that the multiple nodules could be controlled by MCN at HCC team meetings. We also considered MCN for tumors that were more than 5 mm away from the major Glissonean pedicles.

Perioperative morbidities were stratified by severity based on the Clavien-Dindo classification^19^. Major complications were considered as grade III or above. All patients were followed by ultrasound and blood chemistry evaluations every 2–3 months and also dynamic computed tomography or enhanced magnetic resonance every 4–6 months. In case of recurrent tumors, MCN or hepatic resection was planned again based on the same criteria used to select the treatment for the initial tumor regardless of the type of recurrence. However, if surgical approach, such as MCN or hepatic resection, was considered unsuitable to treat multiple recurrences, we performed transcatheter arterial chemoembolization or hepatic arterial infusion chemotherapy. In case of extrahepatic metastases, patients were usually treated with sorafenib or radiation therapy.

### Statistical analysis

Continuous variables are expressed as the median values with ranges. Overall survival (OS) was defined as the interval from surgery to death or the date of the last or most recent follow-up. All patients were followed up until death or June 2019. Recurrence-free survival (RFS) was defined as the interval from surgery to the date of diagnosis of the first recurrence or last follow-up. OS and RFS curves were calculated using the Kaplan–Meier method and compared using the log-rank test. We used a Cox proportional hazards model for univariate and multivariate analyses of prognostic factors related to survival and recurrence. All *p*-values were derived from two-tailed tests with* p* < 0.05 considered as statistically significant. All statistical analyses were performed with EZR (Saitama Medical Center, Jichi Medical University)^20^.

### Compliance with ethical requirements

All procedures were in accordance with the ethical standards of the responsible committee on human experimentation (institutional and national) and the Helsinki Declaration of 1975, as revised in 2008. Informed consent was obtained from all patients included in the study.

## Results

### Clinicopathological characteristics and perioperative outcomes

**Figure 1:**
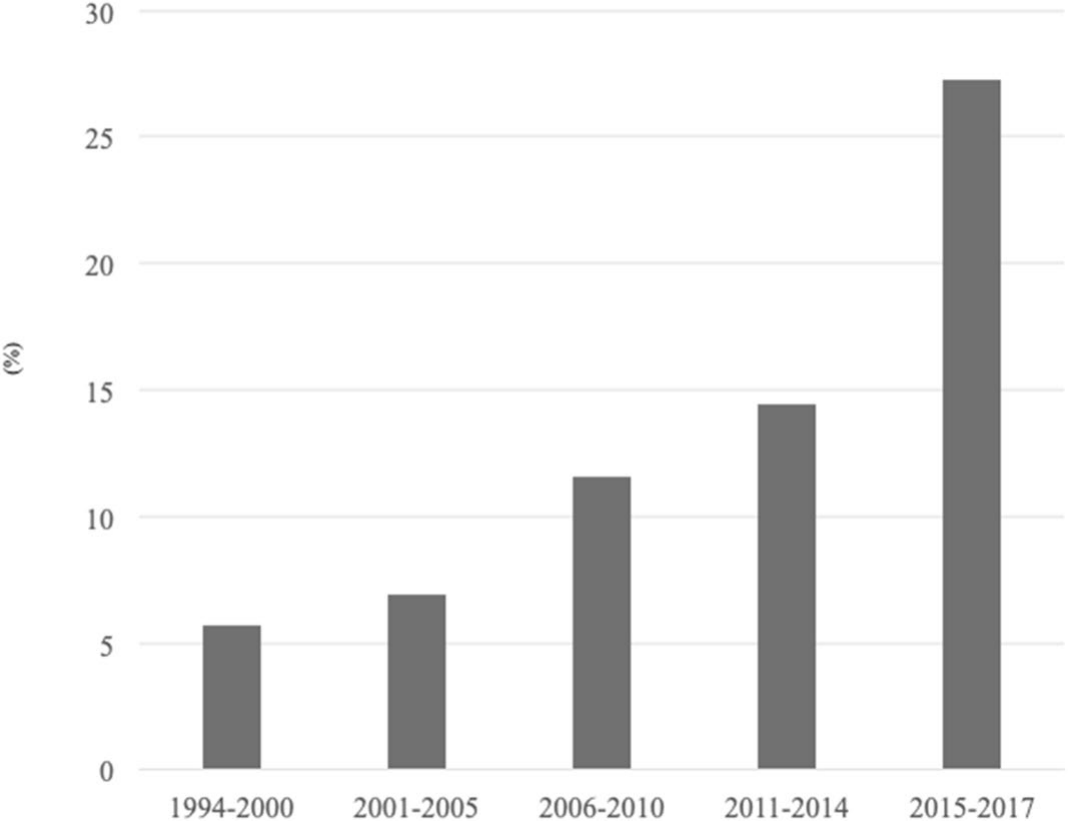
Proportion of elderly patients with HCC at our institute. There was 5.6% (8/141) of patients with HCC aged ≥ 80 years in 1994–2000, 6.9% (16/231) in 2001–2005, 11.5% (40/346) in 2006–2010, 14.4% (26/180) in 2011–2014, and 27.2% (24/88) in 2015–2017. *HCC* hepatocellular carcinoma.

The proportion of HCC patients aged ≥ 80 years was 5.6% (8/141) in 1994–2000 and 27.2% (24/88) in 2015–2017 (Fig. 1). The clinicopathological characteristics are summarized in Table 1. A total of 114 patients met our criteria, including 64 males (56%), with a median age of 82 (80–94) years. Eighty-two (72%) patients had HCV infection. Fourteen patients (12%) had a sustained virological response (SVR) before HCC occurrence. The median maximum tumor size was 26.3 mm (10.5–49), and 60 patients (53%) had a single HCC tumor. The median number of tumor was 1 (1–6).

**Table 1:** Clinicopathological characteristics.

Characteristic	
Age, years, median (range)	82 (80–94)
Sex, male, n (%)	64 (56)
ASA physical status classification 3, n (%)	17 (15)
Alcohol, n (%)	21 (18)
**Concurrent disease**	57 (50)
Diabetes mellitus, n (%)	30 (26)
Cardiovascular disease, n (%)	23 (20)
Hypertension, n (%)	21 (18)
Cerebrovascular disease, n (%)	6 (5)
Renal disease, n (%)	2 (2)
Respiratory disease, n (%)	1 (1)
Dementia, n (%)	1 (1)
Past history of malignant disease	16 (14)
HBs-Ag, n (%)	7 (6)
HCV-Ab, n (%)	82 (72)
Albumin, g/dL, median (range)	3.8 (2.6–4.7)
Total bilirubin, mg/dL, median (range)	0.7 (0.3–2.2)
Prothrombin activity, %, median (range)	86 (56–114)
ICGR15, %, median (range)	19 (4.5–61)
Platelets, /μL, median (range)	13.2 (3.0–28.0)
Child–Pugh class A, n (%)	100 (88)
AFP, ng/mL, median (range)	16.9 (1.1–7,541)
DCP, mAU/mL, median (range)	76.5 (0–25,100)
**Tumor size, mm, median (range)**	26.3 (10.5–49)
≤ 30 mm, n (%)	74 (65)
**Number of tumors, median (range)**	1 (1–6)
Single, n, (%)	60 (53)
2–3, n, (%)	37 (32)
> 3, n, (%)	17 (15)

*ASA* American Society of Anesthesiologists, *ICGR15* indocyanine green retention rate at 15 min, *HBs-Ag* Hepatitis B surface antigen, *HCV-Ab* Hepatitis C virus antibody, *AFP* alpha fetoprotein, *DCP* des-gamma carboxyprothrombin.

### Perioperative outcomes

Perioperative outcomes are summarized in Table 2. The morbidity rate was 11.4% (13/114) [ascites (n = 4), wound infection (n = 3), delirium (n = 3), pleural effusion (n = 2), and intra-abdominal abscess (n = 1)]. The overall major morbidity rate (Clavien-Dindo grade IIIA or above) was 2.6% (3/114) [wound infection (n = 2) and ascites (n = 1)]. There were no cases of mortality.

**Table 2:** Perioperative outcomes.

Characteristics	
**Type of approach for MCN**	
Thoracotomy	63 (55)
Laparotomy	47 (41)
Laparoscopically	4 (5)
Operating time, min, median (range)	94 (39–218)
Blood loss, g, median (range)	7 (0–262)
Intraoperative blood transfusion, n (%)	3 (3)
**Morbidity, n (%)**	13 (11)
Ascites	4 (4)
Wound infection	3 (2)
Delirium	3 (2)
Pleural effusion	2 (1)
Intra-abdominal abscess	1 (1)
**Complication of Clavien-Dindo grade IIIA or above, n (%)**	3 (3)
IIIA	3 (3)
IIIB	0 (0)
IV	0 (0)
V	0 (0)
Mortality, n (%)	0 (0)
Postoperative hospital stay, days, median (range)	12 (5–38)

*MCN* microwave coagulo-necrotic therapy.

### Long-term outcomes

**Figure 2:**
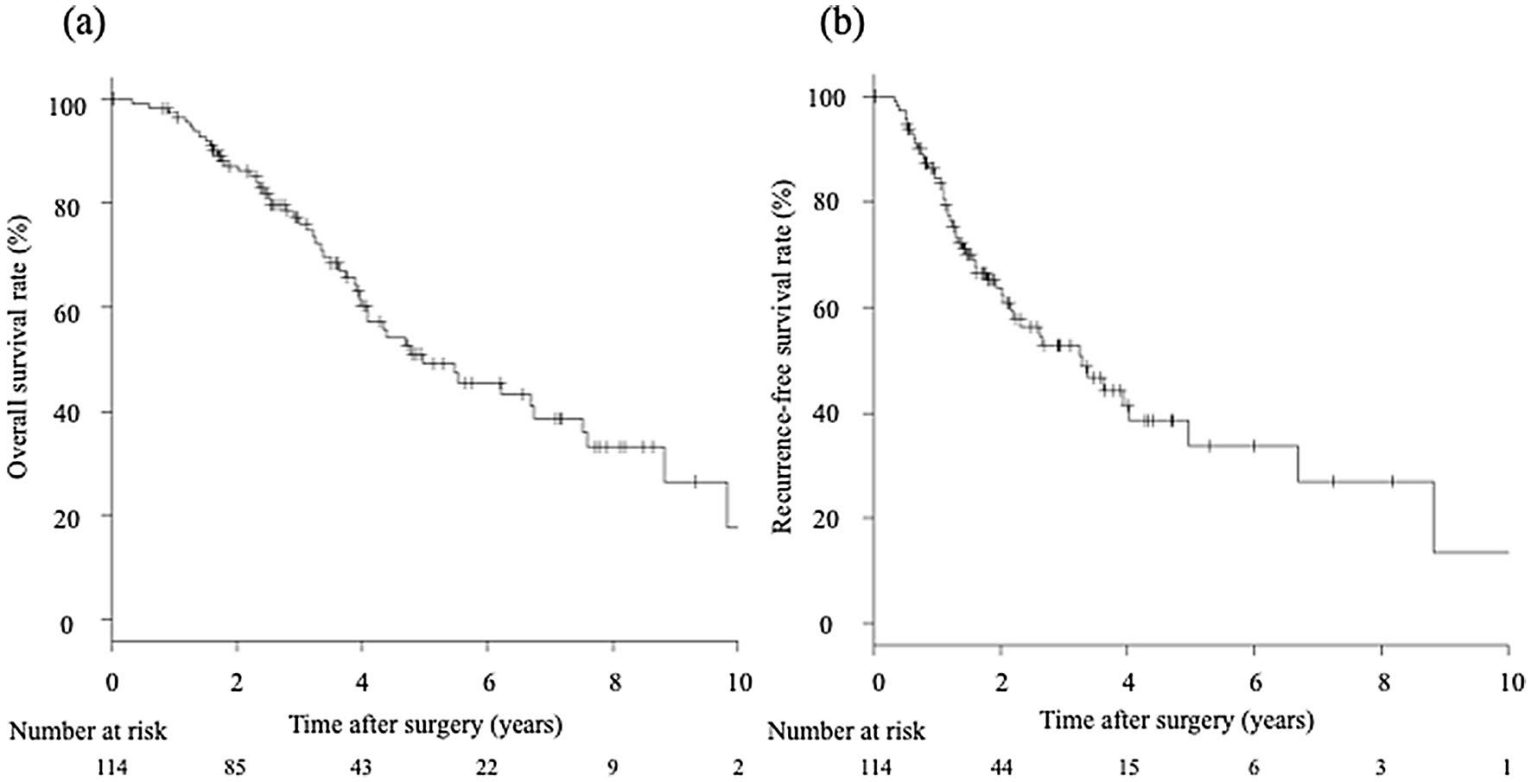
Long-term outcomes of elderly patients with HCC. (**a**) The 1-, 3-, and 5-year OS rates were 97.3%, 76.0%, and 49.2%, respectively. (**b**) The 1-, 3-, and 5-year RFS rates were 84.2%, 44.7%, and 32.5%, respectively. *HCC* hepatocellular carcinoma, *OS* overall survival, *RFS* recurrence-free survival.

The median follow-up time was 40 months (6–135). The prognoses after MCN are summarized in Fig. 2. The 1-, 3-, and 5-year OS rates were 97.3%, 76.0%, and 49.2%, respectively (Fig. 2a). The 1-, 3-, and 5-year RFS rates were 84.2%, 44.7%, and 32.5%, respectively (Fig. 2b). During follow-up, the number of cancer- or liver-related deaths was 50 (44%) and that of other causes of death was 12 (11%). Fifty-nine patients (52%) had recurrences in the whole follow up and four patients (3.5%) had a local recurrence during the first year after surgery. Among recurrence cases, 27 patients underwent MCN, 13 patients underwent transcatheter arterial chemoembolization or hepatic arterial infusion chemotherapy, 11 patients had other treatments, and eight patients had the best supportive care for recurrent HCC.

### Prognostic factors associated with overall survival and recurrence-free survival

**Figure 3:**
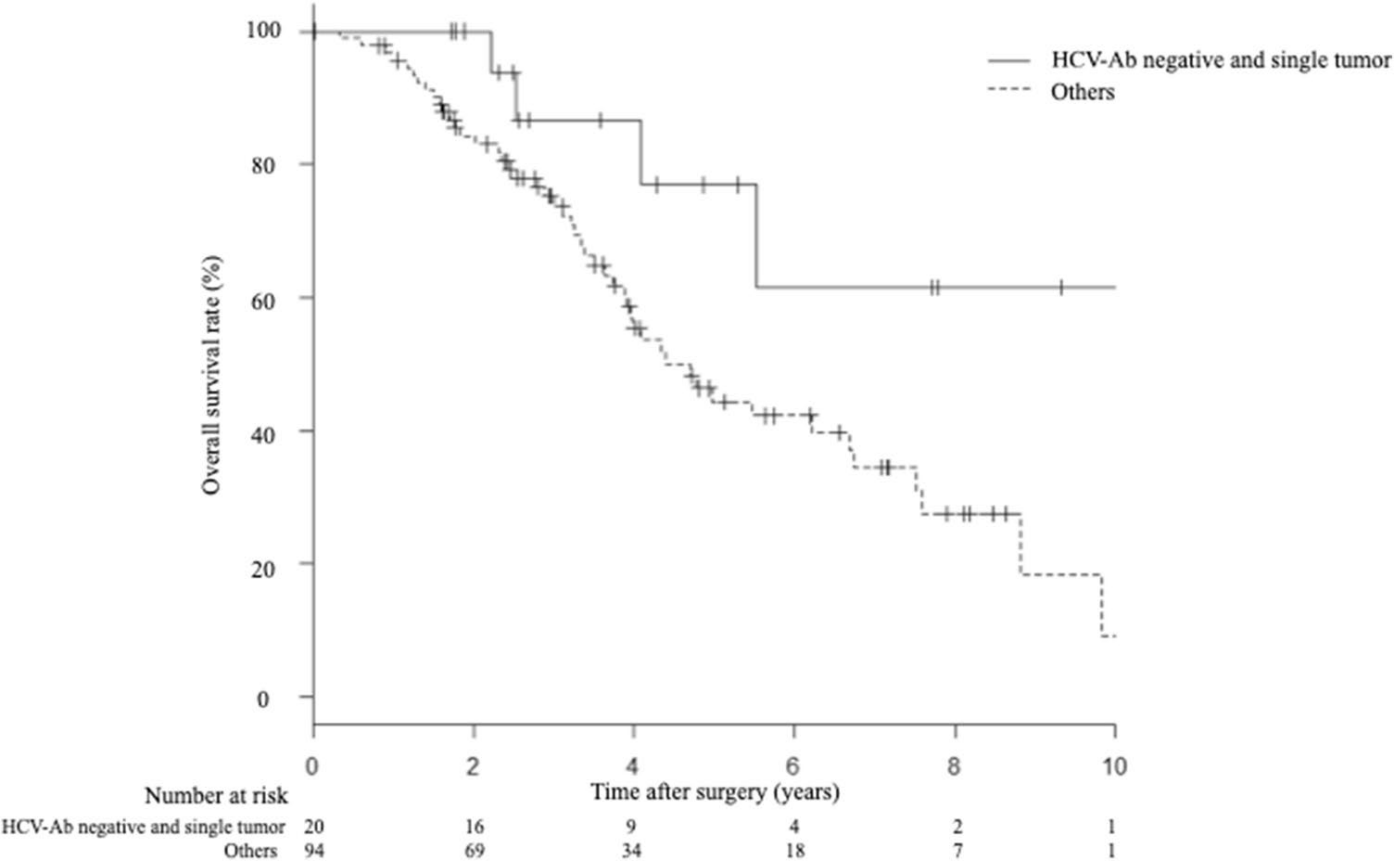
OS compared between patients with HCV-Ab negative and single tumor, and other patients. The 1-, 3-, and 5-year OS rates of patients with HCV-Ab negative and single tumor, and other patients were 100%, 86.5%, and 76.9%, and 96.8%, 73.4%, and 44.4%, respectively (*p* = 0.026). *OS* overall survival, *HCV-Ab* Hepatitis C virus antibody.

Factors associated with OS and RFS were evaluated by univariate and multivariate analyses (Table 3). Multivariate analysis showed that HCV-Ab positivity [hazard ratio (HR), 2.42; 95% confidence interval (CI), 1.11–5.27; *p* = 0.025] and the presence of multiple tumors (HR, 2.11; 95% CI 1.16–3.84; *p* = 0.014) were independent risk factors for OS. Multivariate analyses also showed that HCV-Ab positivity (HR, 3.05; 95% CI 1.32–7.04; *p* < 0.009) and the presence of multiple tumors (HR, 3.10; 95% CI 1.69–5.66;* p* < 0.001) were independent risk factors for RFS. The 1-, 3-, and 5-year OS rates of patients with HCV-Ab negative and single tumor were 100%, 86.5%, and 76.9%, respectively. The 1-, 3-, and 5-year OS rates of others were 96.8%, 73.4%, and 44.4%, respectively. The OS rate of patients with HCV-Ab negative and single tumor was significantly better than that of others (*p* = 0.026) (Fig. 3).

**Table 3:** Prognostic factors associated with overall survival and recurrence-free survival.

Characteristics	Overall survival						Recurrence-free survival					
	Univariate analysis			Multivariate analysis			Univariate analysis			Multivariate analysis		
	HR	95% CI	*p*-value	HR	95% CI	*p*-value	HR	95% CI	*p*-value	HR	95% CI	*p*-value
Sex (male)	0.81	0.42–1.54	0.527				0.96	0.51–1.83	0.921			
ASA (class 3)	1.73	0.71–4.19	0.219				1.51	0.63–3.63	0.348			
Past history of malignant disease ( +)	1.86	0.78–4.41	0.156				1.49	0.62–3.60	0.366			
HCV-Ab ( +)	2.29	1.01–5.20	0.046	2.42	1.11–5.27	0.025	2.68	1.14–6.28	0.023	3.05	1.32–7.04	0.009
Child–Pugh (Grade B)	1.29	0.47–3.50	0.615				1.75	0.66–4.65	0.258			
Platelet count (× 10^4^/µL) (≤ 10)	1.17	0.56–2.46	0.671				0.85	0.41–1.78	0.684			
ICGR15 (%) (> 15)	1.33	0.65–2.71	0.422				1.91	0.92–3.97	0.080			
AFP (> 20)	1.35	0.65–2.79	0.415				1.46	0.68–3.12	0.320			
DCP (> 40)	1.18	0.57–2.45	0.643				1.37	0.64–2.92	0.411			
Maximum tumor size (mm) (> 30)	1.57	0.79–3.08	0.190				1.60	0.84–3.06	0.150			
Number of tumors (multiple)	2.35	1.17–4.73	0.015	2.11	1.16–3.84	0.014	3.22	1.59–6.51	0.0011	3.10	1.69–5.66	< 0.001

*HR* hazard ratio, *CI* confidence interval, *ASA* American Society of Anesthesiologists, *HCV-Ab* hepatitis C virus antibody, *ICG-R15* indocyanine green retention rate at 15 min, *AFP* Alpha-fetoprotein, *DCP* Des-γ-carboxy prothrombin.

## Discussion

This study investigated the feasibility and safety of surgical microwave ablation in elderly HCC patients (≥ 80 years). Our study revealed that surgical microwave ablation was performed feasibly and safely in elderly patients with primary HCC, who had a 5-year OS rate of 49.2%. Although several studies have reported favorable effects of hepatic resection and RFA, the feasibility and safety of microwave ablation in elderly HCC patients has not been reported. To the best of our knowledge, this is the first study to clarify the perioperative and oncological outcomes of microwave ablation in elderly patients with HCC.

According to Japanese surveys of primary liver cancer, the age of HCC patients is increasing^1^. In our department, the proportion of elderly patients (≥ 80 years) with HCC is also increasing. About 27% of patients with HCC were aged ≥ 80 years in 2015–2017. Hepatic resection is generally considered as the first line treatment, even in elderly HCC patients with normal liver functions and good general conditions. Some previous studies of long-term follow-ups after hepatic resection in elderly patients have reported 5-year OS rates of 35.5–59.5%^3,4,21^. However, elderly patients are a higher risk group than younger patients because of comorbidities and are therefore considered as a high risk group for major surgery^22^. A previous report revealed that hepatic resection was performed for only 31% of elderly HCC patients (≥ 75 years) among primary treatments^2^. It was suggested that many elderly HCC patients could not be candidates for hepatic resection because of their poor general status, and the indication of hepatic resection was limited to a carefully selected elderly population.

Local ablation therapies, such as RFA and microwave ablation, are considered to be reasonable treatments instead of liver resection. Among local ablation therapies, RFA is the most widely used. Several previous studies have shown that RFA achieves similar survival outcomes to hepatic resection for early stage HCC^23,24^. According to the management guidelines of the European Association for the Study of the Liver and the American Association for the Study of Liver Diseases, RFA is recommended as a treatment modality for early stage HCC with hepatic resection and liver transplantation based on liver functions^7,8^. The efficacy of RFA in elderly HCC patients has also been reported with 5-year OS rates of 49.7–57%^9,25^. In a recent retrospective comparative study, there were no significant differences between hepatic resection and RFA for elderly HCC patients (≥ 65 years) with 5-year survival of 51.9% and 55.2%, respectively^26^.

In recent years, the treatment efficacy of microwave ablation has been reported^6^. There are theoretically some advantages to microwave ablation compared to RFA. Microwave ablation has large ablation zones and higher intratumoral temperature, which contributes complete coagulative necrosis both tumor and a rim of normal liver tissue^10^. Several previous studies have also reported that microwave ablation showed comparative results compared to RFA for primary HCC when comparing local recurrence rates and long-term survival with 5-year OS of 43–60%^11–14,27^. We have performed surgical microwave ablation, termed microwave coagulo-necrotic therapy (MCN), as one of the treatment modalities of HCC patients for more than 20 years, and previously reported its feasibility, safety, and long-term outcomes^14–17^. In our recent propensity score matching analysis, we clarified that MCN provided almost equivalent OS and RFS as hepatic resection for single HCC of < 5 cm with less invasive perioperative outcomes^15^. It is our firm belief that MCN is a feasible treatment option, especially for patients with impaired liver functions and elderly patients.

In this study, we analyzed the oncological outcomes of MCN for elderly HCC patients with a 5-year OS rate of 49.2%. This result is comparable with that of previous reports for both hepatic resection and RFA in elderly patients with HCC. The local recurrence rate at 1-year was also equivalent compared to previous report^14^. We think that MCN could provide good loco-regional control even in elderly population. We also analyzed the perioperative outcomes of surgical microwave ablation in elderly patients. The major morbidity rate was 2.6% [wound infection (n = 2) and ascites (n = 1)] with no procedure-related deaths in this study. These findings suggest that surgical microwave ablation is a safe and feasible procedure for elderly HCC patients.

Another important finding in this study was that we revealed several factors independently associated with long-term outcomes of an elderly population. In our multivariate analysis, HCV-Ab positivity and multiple tumors were independent prognostic factors for OS and RFS. In previous studies of elderly HCC patients, an ASA grade of > 2, tumor size, and tumor markers were associated with increased morbidity and reduced survival^2,9,26,28^. In our study, an ASA grade of > 3 did not affect the long-term outcomes. This can be attributed to the fact that there was a limited number of ASA grade > 3 patients in this study. We also analyzed the long-term outcomes according to prognostic factors. Patients with HCV-Ab negative and single tumor had better long-term outcomes compared with other patients (*p* = 0.026). It is conceivable that patients with HCV-Ab negative and single tumor are good candidates for surgical microwave ablation, especially the elderly population. Further studies are needed to precisely determine the indication for surgical microwave ablation of elderly patients with HCC.

This study has several limitations. First, it is based on a single center review and has a limited number of patients. Second, there is the potential for selection bias because of the retrospective design. Lastly, this study did not consider SVR of HCV-Ab-positive patients. The introduction of direct-acting antiviral agents (DAA) resulted in an increase of patients with HCV who achieve SVR in recent years^29^. We recently reported the importance of achieving SVR before HCC occurrence to reduce recurrence and to improve long-term outcomes after MCN in HCV-related HCC patients^30^. As the number of patients with HCV who achieve SVR increase, further analyses would be required to examine the long-term outcomes of HCV-related patients who achieve SVR in the future. Despite these limitations, the results of this study may be clinically informative and may an indicator for the future analysis of the treatments for elderly HCC patients.

In conclusion, we analyzed the feasibility and safety of microwave ablation in elderly patients with HCC. Our results suggested that surgical microwave ablation was a safe and feasible treatment for elderly patients. In particular, elderly patients with HCV-Ab negative and single tumor were expected to have better long-term outcomes after surgical microwave ablation. Therefore, microwave ablation can be a useful treatment modality for elderly patients with HCC.

## Abbreviations

HCC: Hepatocellular carcinoma
HCV-Ab: Hepatitis C virus antibody
RFA: Radiofrequency ablation
MCN: Microwave coagulo-necrotic therapy
ASA classification: American Society of Anesthesiologists classification
HBs-Ag: Hepatitis B surface antigen
ICG-R15: Indocyanine green retention rate at 15 min
AFP: Alpha-fetoprotein
DCP: Des-γ-carboxy prothrombin
OS: Overall survival
RFS: Recurrence-free survival
SVR: Sustained virological response
HR: Hazard ratio
CI: Confidence interval
DAA: Direct-acting antiviral agents
